# Hydrorelease Within the Paraneural Sheath: A Cadaveric Study

**DOI:** 10.3390/jfmk11020199

**Published:** 2026-05-17

**Authors:** Kousuke Shiwaku, Carmelo Pirri, Hidenori Otsubo, Andrea Porzionato, Rikiya Itagaki, Daiki Nishikawa, Tomoaki Kamiya, Daisuke Suzuki, Hiroyuki Takashima, Makoto Emori, Atsushi Teramoto, Carla Stecco

**Affiliations:** 1 Department of Neuroscience, University of Padova, 35122 Padova, Italy; shiwaku1008@gmail.com (K.S.); carmelop87@hotmail.it (C.P.); andrea.porzionato@unipd.it (A.P.); 2 Department of Orthopedic Surgery, Sapporo Medical University School of Medicine, Sapporo 060-8556, Japan; rikiya.i.0829@gmail.com (R.I.); dnishikawa0110@gmail.com (D.N.); tkamiya0606@gmail.com (T.K.); memori@sapmed.ac.jp (M.E.); teramoto.atsushi@gmail.com (A.T.); 3 Sapporo Sports Clinic, Sapporo 060-0041, Japan; otsubo-orth@umin.ac.jp; 4 Department of Rehabilitation, Faculty of Health Science, Hokkaido Chitose Collage of Rehabilitation, Chitose 066-0055, Japan; daisuke@sapmed.ac.jp; 5 Graduate School of Health Sciences, Sapporo Medical University, Sapporo 060-0812, Japan; hirotakashima@pop.med.hokudai.ac.jp

**Keywords:** hydrorelease, hydrodissection, injection, intra-paraneural sheath, layers

## Abstract

**Background:** Definitive quantification of fluid spread within the paraneural sheath (PNS) but external to the epineurium during hydrorelease (HR)-like procedures is lacking. We aimed to investigate the spread of low-volume HR within the intra-PNS surrounding the sciatic, tibial, and common peroneal nerves using human cadaveric specimens. **Methods:** HR with 2.5 mL of dye-mixed saline was performed under ultrasound guidance into the intra-PNS of seven lower limbs from four fresh-frozen cadavers. Dye spread was quantified by measuring longitudinal distance and circumferential dispersion, followed by anatomical dissection within 1 min of injection. **Results:** All injections demonstrated consistent longitudinal spread along the intra-PNS layer without intraneural infiltration. The mean spread distances were 10.63 ± 3.66, 9.97 ± 3.60, and 8.36 ± 3.04 cm in the sciatic, tibial, and common peroneal nerves, respectively, indicating no significant differences. An opposite-side circumferential spread was observed in all cases, with mean scores indicating mild-to-moderate extension. **Conclusions:** Low-volume HR selectively spreads within the intra-PNS layer, suggesting that this anatomical layer is a structurally valid and reproducible target for perineural injection techniques.

## 1. Introduction

Hydrorelease (HR) is a common minimally invasive injection technique targeting perineural and interfascial layers [1,2,3,4]. This method involves the ultrasound-guided administration of small volumes (1–10 mL) of normal saline into loose connective tissues surrounding nerves or between fascial layers to restore interfascial gliding and alleviate pain and tightness. Conversely, hydrodissection (HD) is performed by injecting large fluid volumes (10–50 mL) of pharmacologically active fluid mixtures of anesthetics (e.g., bupivacaine and lidocaine), corticosteroids, and saline to mechanically separate fascial layers [5,6,7]. The clinical outcomes of significant pain reduction after HR have been reported [1,2]. However, studies on the mechanisms underlying pain relief or range of motion improvement are lacking. To date, publications elucidating the precise mechanism of HR are scarce; only one biomechanical study has demonstrated that HR reduces fascial gliding resistance [3].

**Figure 1:**
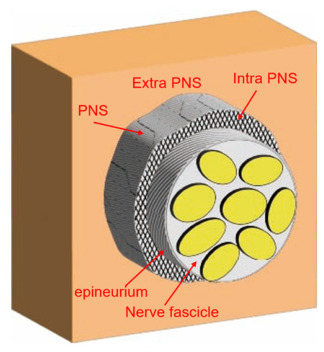
Schematic representation of the PNS around a peripheral nerve. Anatomical organization of a peripheral nerve, comprising nerve fascicles encased by the epineurium and the PNS that surrounds it. The intra-PNS layer is defined as the region between the epineurium and the PNS, whereas the extra-PNS lies external to the PNS. PNS: paraneural sheath.

In clinical practice, HR targeting peripheral nerves is performed within the paraneural sheath (intra-PNS) or outside the sheath (extra-PNS) (Figure 1). The intra-PNS approach involves injecting fluid into the layer between the epineurium and the PNS, whereas the extra-PNS approach targets areas external to the PNS [3,4]. These anatomical distinctions are crucial for understanding the spread of the injectate and its potential therapeutic effects.

In a cadaveric study involving the extra-PNS HR technique, the dye injected between the aponeurotic fascia (APF) and the epimysium (EPI) remained confined to the interfascial layer, suggesting a high degree of structural selectivity [4]. Similarly, 1 mL of saline injected between two EPI layers spread effectively within the target layer, supporting the reproducibility and anatomical specificity of low-volume injections [8]. Moreover, Shiwaku et al. [3,4] showed that injecting 2.5 mL of saline into the loose connective tissue between the APF and EPI or between two adjacent EPI layers significantly reduced gliding resistance in fresh frozen cadaveric specimens [3]. These findings suggest that the mechanism of action of HR in extra-PNS targets involves restoring fascial gliding by modulating mechanical stiffness through targeted fluid delivery.

In contrast, for intra-PNS, the anatomical characteristics of even fluid distribution following injection into the intra-PNS (within the PNS but external to the epineurium) remain poorly understood. To date, anatomical studies have not definitively quantified fluid spread within this specific fascial layer during HR-like procedures. Moreover, regardless of the injection technique (HD or regional nerve block), whether small-volume injections consistently remain confined within the loose connective tissue layer of the intra-PNS is understudied. Consequently, the structural behavior and potential clinical implications of fluid delivery into this anatomical layer remain largely unexplored.

Therefore, we aimed to anatomically evaluate the longitudinal quantitative spread of a 2.5 mL dye-saline solution injected at a single site within the PNS and external to the epineurium of the sciatic, tibial, and common peroneal nerves, using fresh-frozen cadavers. This pilot anatomical study was designed to simulate the clinical HR procedure, focusing on the intra-PNS layer.

We hypothesized that a small fluid volume would selectively distribute within the loose connective tissue of the intra-PNS, achieving a relatively wide longitudinal spread while remaining confined to this layer without leaking into non-targeted layers.

## 2. Materials and Methods

### 2.1. Specimen Preparation

This cadaveric study was designed to anatomically investigate the spatial distribution of fluid following HR into the intra-PNS. All procedures were conducted according to international guidelines and relevant legislation governing the use of human cadaveric specimens for anatomical research. The retrieval, handling, use, and disposal of the cadaveric specimens were conducted under the institutional ethical framework of the Body Donation Program of the Institute of Human Anatomy, University of Padova.

At the popliteal region, the sciatic nerve divides into the tibial nerve and the common peroneal nerve. The tibial nerve continues distally through the popliteal fossa, whereas the common peroneal nerve courses dorsolaterally toward the fibular head. In the posterior knee region, these nerve structures lie immediately beneath the popliteal fascia/membrane and represent relatively superficial structures beneath this layer. These topographical relationships were considered when standardizing the ultrasound transducer positions and injection levels for each target nerve.

In this study, the PNS was considered a macroscopically identifiable connective tissue sheath surrounding the sciatic nerve and its major branches. Around the sciatic bifurcation, the tibial and common peroneal components may share a common paraneural connective tissue space before separating into branch-specific sheath structures. However, the present study was not designed to determine the exact histological continuity or branching pattern of the PNS between the sciatic trunk and each branch. Therefore, dye spread was evaluated within macroscopically identifiable perineural connective tissue planes.

**Figure 2:**
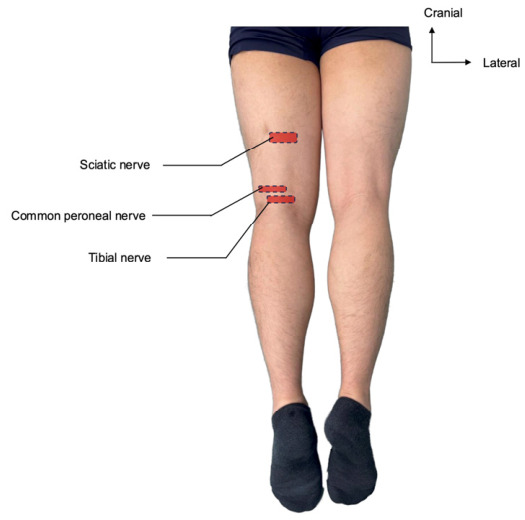
Posterior view of lower limb showing standardized transducer positions (red rectangles) and target nerves. Orientation arrows indicate cranial and lateral. Rectangles mark: mid-thigh for the sciatic nerve, at the joint line for the tibial nerve, and 2 cm proximal to the joint line for the common peroneal nerve.

Seven lower limbs (from four fresh-frozen human cadavers) were used in this study. The specimens were donated to the Institute of Human Anatomy of the University of Padova through the institutional Body Donation Program. All specimens exhibited no apparent signs of prior trauma or surgical intervention in the lower extremities. The four donors comprised two females (aged 74 and 85 years) and two males (aged 72 and 77 years), with a mean age of 77.0 years (range, 72–85 years). Three cadavers contributed both lower limbs to the analysis, while one cadaver (the 72-year-old male) contributed only the left lower limb, yielding seven specimens in total. Detailed information regarding body mass index, cause of death, and comorbidities was not available through the institutional Body Donation Program of the University of Padova, which discloses only the donor’s age and sex to investigators. Specimens with obvious lower-limb trauma, previous orthopedic surgery, marked scarring, or gross pathological changes around the target nerves were excluded. The target lower-limb regions were not dissected or autopsied before the ultrasound-guided injections. No artificial replenishment of tissue fluid or tissue turgor was performed. Each cadaver was thawed at room temperature for at least 24 h before the experimental procedures and placed in the supine position. During the experiment, the tissues were kept moist with saline-soaked gauze to minimize dehydration-related changes and maintain structural and textural fidelity. The sciatic, tibial, and common peroneal nerves were selected as target structures for analysis. The injection levels were standardized as follows: at the mid-thigh level (center of femur) for the sciatic nerve, at the joint line for the tibial nerve, and 2 cm proximal to the joint line for the common peroneal nerve (Figure 2). Probe positions were confirmed using palpable surface anatomical landmarks—including the ischial tuberosity, the medial and lateral femoral epicondyles, the popliteal crease, and the fibular head—prior to ultrasound application, ensuring reproducibility of transducer placement on a real-life lower limb.

### 2.2. Ultrasound-Guided Injection

**Figure 3:**
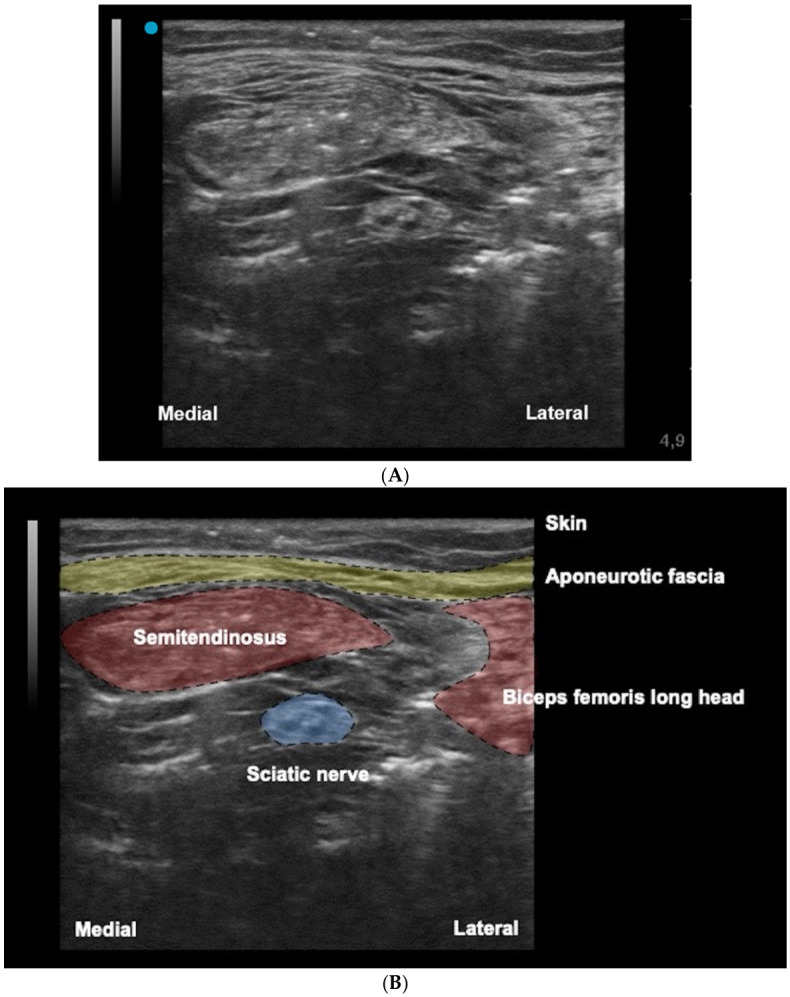
Short-axis ultrasound image of the sciatic nerve at the injection level. (**A**) Unannotated ultrasound image. (**B**) Annotated image corresponding to panel (**A**). The skin, aponeurotic fascia, semitendinosus muscle, biceps femoris long head, and sciatic nerve are labeled. Medial and lateral orientations are also indicated.

An ultrasound-guided injection was performed for each nerve. The local anatomy of the sciatic nerve and surrounding structures at the injection level is shown in Figure 3 (panel A: unannotated; panel B: annotated). Before needle insertion, the operator (first author) thoroughly explored the course of the nerve to identify a region where the loose connective tissue within the PNS was clearly visible on ultrasound. The PNS was defined as a thin layer of loose connective tissue situated between the epineurium and the APF. A 25-gauge, 40 mm needle was introduced using an in-plane technique under continuous ultrasound visualization. Ultrasound imaging was performed using a 6–15 MHz linear transducer (Edge II, Sonosite, FUJIFILM, Seattle, WA, USA).

Intra-PNS needle placement was defined as continuous visualization of the needle tip within the hypoechoic potential space between the hyperechoic epineurial surface and the outer PNS-like connective tissue layer. Correct placement was further supported by the formation of a crescent-shaped hypoechoic fluid layer around the nerve during slow injection, without intraneural swelling, fascicular separation, or marked expansion of the nerve itself. The final location of the injectate was confirmed by immediate anatomical dissection.

The injection site was standardized to the middle-to-lower fourth depth of each nerve. The needle was advanced carefully into the hypoechoic layer between the PNS (hyperechoic) and the epineurium (hyperechoic). Subsequently, 2.5 mL of a saline and dye mixture was slowly injected into a single point within the intra-PNS. During injection, fluid spread was monitored in real time mainly in the short-axis view to confirm expansion within the intended perineural plane and to exclude obvious intraneural expansion. Longitudinal spread along the nerve axis was subsequently quantified by anatomical dissection rather than by continuous real-time ultrasound tracking along the entire nerve length. The insertion depth and angle were standardized optimally across all specimens to minimize variability in the injection technique. To ensure anatomical accuracy of the labeled structures depicted in Figure 3B, the labels were verified not only on the selected still image but also across the continuous cine sequence to ensure spatial consistency of the anatomical structures, and were independently cross-checked among co-authors with expertise in musculoskeletal ultrasound anatomy.

### 2.3. Confirmation by Dissection

A skin incision was made within 1 min after injection, and the target nerve was carefully exposed while preserving the surrounding tissues. The extent of the longitudinal spread of the dye solution along the injected side of the nerve was subsequently measured in centimeters using the maximum visible distance coloration as the outcome parameter.

In addition, circumferential dye dispersion to the side opposite the injection point within the same nerve circumference was evaluated using a three-grade visual scoring system: Grade 1, no dye spread to the opposite side; Grade 2, spread < 2 cm; and Grade 3, spread ≥ 2 cm. In this context, “opposite side” refers to the side opposite the injection point within the circumference of the same injected nerve, not to the contralateral lower limb. This classification was established to objectively define the degree to which the injectate extended beyond the target side of the nerve within the intra-PNS layer.

All dissections were primarily performed by a co-author (C.S.) with extensive experience in fascia anatomy. Dissection proceeded stepwise with maximal preservation of fascial planes (APF, EPI, epineurium, and PNS), and layer boundaries were identified before proceeding to the next step. In addition, an exploratory 24 h post-injection evaluation was performed for the common peroneal nerve. Using the same ultrasound-guided protocol as in the primary experiments, 2.5 mL of dye-mixed saline was injected into the intra-PNS. The specimen was then left undisturbed, and anatomical dissection was performed 24 h later. Longitudinal spread and circumferential dispersion were evaluated using the same methods as in the primary experiment. The 24 h specimen was dissected using identical stepwise procedures, maintaining maximal preservation of the aponeurotic fascia, epimysium, epineurium, and paraneural sheath. This delayed time point was selected to assess whether the layer specificity of HR is preserved beyond the immediate post-injection phase typically evaluated in cadaveric studies, thereby better simulating the time course relevant to clinical hydrorelease applications.

### 2.4. Statistical Analysis

To compare the longitudinal spread of the injectate on the injected side among the three nerve groups (sciatic, tibial, and common peroneal nerves), a one-way analysis of variance was performed, followed by Tukey’s post hoc test for multiple comparisons.

For the semi-quantitative grading of circumferential spread to the side opposite the injection point within the same nerve (Grades 1–3), non-parametric analysis was conducted using the Kruskal–Wallis test. If a significant difference was found, Fisher’s exact test was subsequently applied to examine intergroup differences.

A formal a priori sample size calculation was not performed because this was an exploratory cadaveric anatomical study designed to evaluate the feasibility and distribution pattern of low-volume injectate within the intra-PNS rather than to test a predefined clinical hypothesis. The number of specimens was determined based on the availability of fresh-frozen human cadavers and the ethical and practical constraints inherent to cadaveric research.

All analyses were performed using the Statistical Package for Social Sciences (version 28.0; IBM Corp.; Armonk, NY, USA), with the level of statistical significance set at *p* < 0.05.

## 3. Results

**Figure 4:**
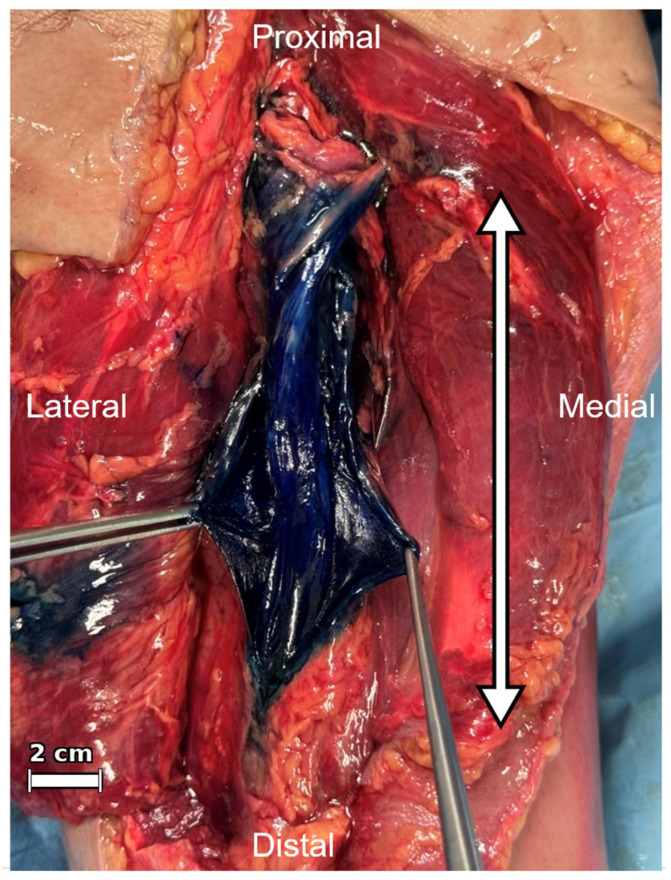
Representative macroscopic image showing longitudinal dye spread along the sciatic nerve after ultrasound-guided injection into the intra-PNS. The white double-headed arrow indicates the measured longitudinal spread in this specimen (14.8 cm). A centimeter scale bar, calibrated to the measured anatomical distance in this representative specimen, is shown in the lower left. PNS: paraneural sheath.

In all specimens, the injected dye exhibited a longitudinal spread within the PNS along the external surface of the epineurium (within the intra-PNS). The injectate predominantly extended linearly through the loose connective tissue of the intra-PNS, with only minimal extravasation beyond the extra-PNS. No cases of intraneural (within-nerve) penetration were noted. Figure 4 presents a representative image of dye distribution around the sciatic nerve. In all cases, the spread followed the layer of the intra-PNS, with no instances of excessive leakage into adjacent structures or escape beyond the intra-PNS layer.

**Figure 5:**
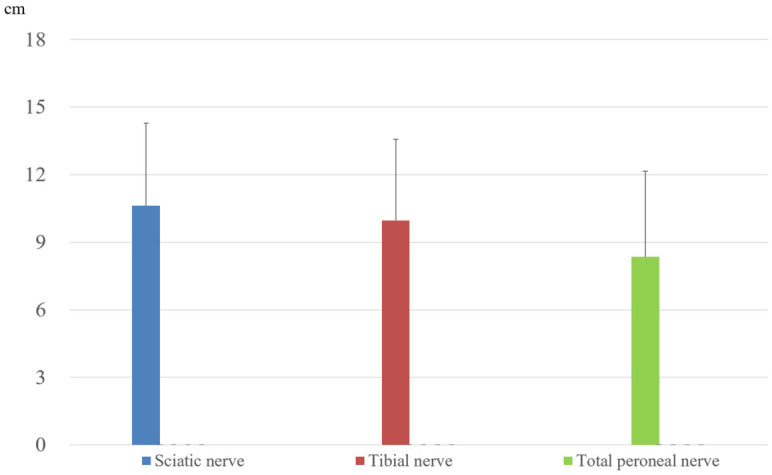
Mean longitudinal spread of injectate within the intra-PNS. The mean longitudinal spread (±SD) of dye-mixed saline (2.5 mL) following ultrasound-guided HR into the intra-PNS of the sciatic, tibial, and common peroneal nerves. No significant differences were observed among the three nerves.

The mean longitudinal spreads on the injected side were 10.63 ± 3.66, 9.97 ± 3.60, and 8.36 ± 3.04 cm for the sciatic, tibial, and common peroneal nerves, respectively (Figure 5). No statistically significant differences were observed; therefore, these data should be interpreted as exploratory rather than confirmatory.

Regarding circumferential dye spread to the side opposite the injection point within the same nerve, the mean visual scoring values were 1.64 ± 0.50, 1.60 ± 0.52, and 1.64 ± 0.50 for the sciatic, tibial, and common peroneal nerves, respectively (Table 1). These values indicate a consistent degree of circumferential dispersion without significant intergroup differences.

The proportion of cases (%) showing no spread, <2 cm spread, and ≥2 cm spread to the side opposite the injection point within the same nerve circumference, assessed visually after ultrasound-guided hydrorelease (2.5 mL) into the intra-paraneural sheath of the sciatic, tibial, and common peroneal nerves. “Opposite side” indicates the side opposite the injection point within the circumference of the same injected nerve, not the contralateral lower limb. No significant differences were observed among the three nerves.

In the exploratory evaluation performed 24 h after injection around the common peroneal nerve, the dye remained distributed within the intra-PNS and demonstrated a maximum longitudinal spread of 13.9 cm along the nerve axis. Circumferential spread to the side opposite the injection point within the same nerve exceeded 2 cm, and no intraneural penetration was identified. These findings were consistent with the distribution pattern observed in the primary experiments.

## 4. Discussion

Until recently, most anatomical studies investigating the distribution of injectate in HR have primarily focused on extra-PNS layers, such as interfascial layers between the APF and EPI [4,8]. No previous studies have considered the quantitative analysis of fluid spread following HR targeting the intra-PNS.

HD has a long-standing use in clinical practice and has been investigated in several anatomical studies; however, the anatomical descriptions of the injection layer are often ambiguous, commonly referring to the layers as “nerve-adjacent,” “perineural,” or “interfascial” regions without clear delineation of whether the injectate was confined to the intra-PNS layer [9,10,11].

HD involves the administration of relatively abundant fluid (10–50 mL), often combined with pharmacological agents such as local anesthetics or corticosteroids, to separate fascial or perineural tissue planes through mechanical force. In contrast, HR is defined as a structurally selective injection of small volumes (1–10 mL) of non-pharmacologic fluid, such as saline, into a defined loose connective tissue layer. This approach emphasizes the concept and precise control of the target layer and injection volume, making HR a fundamentally different technique from HD.

To our knowledge, our study is the first to quantitatively demonstrate how small-volume fluid distributes within the intra-PNS under HR-mimicking conditions. While there are previous cadaveric studies on perineural injections [12], none have quantified the spread of small-volume saline injections within the intra-PNS layer.

Our findings indicate that in an intact intra-PNS layer, a 2.5 mL injection results in a longitudinal spread of approximately 9 cm. This observation may serve as a reference for future evaluations, whereby a significantly shorter spread, such as 3 cm under the same injection conditions, could suggest pathological structural alterations, such as fibrosis or compartmental constriction.

As previously noted, few studies have explicitly described the exact injection layer, particularly with reference to the PNS and whether the procedure was conducted within (intra-PNS) or outside (extra-PNS) this structure [13,14]. There is increasing attention on the structural characteristics of the PNS and its potential role in peripheral nerve mobility and pain pathophysiology. Notably, anatomical studies on carpal tunnel syndrome have suggested that reductions in paraneural fat and thickening of the sheath significantly restrict nerve excursion, contributing to chronic neural dysfunction [15]. These findings imply that the layer between the PNS and the epineurium may not merely function as a passive anatomical space but rather as a structurally and pathologically significant interface.

A comparable anatomical investigation was conducted by Andersen et al. [12], who injected a curable resin rather than saline into a cadaveric model. Their study demonstrated that the PNS constitutes a distinct anatomical structure, distinguishable from that of the epineurium. Additionally, they reported that injecting 10 mL of resin into this intra-PNS produced a longitudinal spread of approximately 10–15 cm. However, they noted certain limitations, including the inability to achieve circumferential dispersion around the nerve and the inherently high viscosity of the resin, which may have influenced the spread characteristics [12].

In clinical contexts, Karmakar et al. [16] provided compelling ultrasound evidence from four cases of sciatic nerve block, wherein the injectate, ropivacaine, was delivered into the subparaneural compartment, a layer topographically consistent with the intra-PNS defined in this study. Their study vividly illustrated a laminar and circumferential spread of the anesthetic within this fascial plane, encompassing the tibial and common peroneal branches of the sciatic nerve. Notably, this compartmentalized distribution caused a more rapid onset and more complete sensory blockade than traditional approaches.

These previous findings support the feasibility of HR as a structurally selective intervention within the intra-PNS from anatomical and clinical perspectives. HR involves using a non-pharmacologic saline injection, and our results suggest that an injection volume of approximately 2.5 mL or slightly more is sufficient for a circumferential spread around the nerve within the intra-PNS space. This volume appears appropriate for maintaining targeted distribution, emphasizing the anatomical validity of HR as a low-volume, layer-specific technique.

Future studies involving in vivo animal models are warranted to investigate how fluid injected into the intra-PNS distributes under dynamic physiological conditions. In addition, a systematic evaluation of procedural variables such as injection pressure, volume, needle gauge, and angle of insertion will be essential to validate the structural feasibility and reproducibility of HR within this anatomical layer. It is particularly crucial to determine how intra-PNS injections targeting loose connective tissue correlate with clinical outcomes, including pain relief and improved mobility. Elucidating this relationship will be critical for refining the understanding of HR and expanding its therapeutic applications.

In this cadaveric study, the injection of 2.5 mL of saline into the intra-PNS consistently produced a longitudinal spread of the injectate across all specimens, with mean distribution lengths ranging approximately 8–11 cm, depending on the nerve. The spread remained largely confined within the intra-PNS layer, with minimal leakage into the surrounding layers and no intraneural penetration. These findings emphasize that HR functions as a structurally selective intervention when performed within the intra-PNS layer. No statistically significant differences were observed among the three nerves concerning longitudinal spread or the degree of circumferential spread to the side opposite the injection point within the same nerve, suggesting that small-volume HR produces a relatively consistent distribution pattern across different peripheral nerves.

We visualized the distribution of fluid following low-volume injection within the intra-PNS using fresh-frozen cadaveric specimens, providing anatomical insights into the underlying mechanism of HR. However, some limitations should be acknowledged. First, the specimens were anatomically normal older individual cadavers that did not exhibit pathological changes, such as impaired fascial gliding commonly observed in clinical cases. The extent to which such pathological tissue characteristics may influence the distribution pattern of the injected fluid should be clarified in future investigations. Second, we used dye diffusion for visualization, offering a primarily qualitative assessment. Biomechanical parameters such as interfascial gliding resistance were not measured. For a comprehensive understanding and optimization of the HR technique, future studies should incorporate quantitative biomechanical or biological evaluations. Third, cadaveric models lack physiological dynamics such as blood flow, tissue pressure, and edema. Therefore, the diffusion behavior of an injectate may differ from that in living tissue. Fourth, our study was limited to static anatomical dissection; fluid behavior during dynamic conditions, such as muscle contraction or joint movement, was not assessed. A proposed mechanism of HR in the extra-PNS context involves restoring fascial gliding; therefore, it is imperative to investigate how fluid distributes within the intra-PNS layer during movement or mechanical loading. Fifth, this study demonstrated that small-volume injections selectively and extensively spread within the intra-PNS layer; however, the injectate is not always confined to this layer in clinical practices. In many cases, fluid is intentionally or unintentionally delivered into intra- and extra-PNS layers. This dual distribution should be considered when interpreting the anatomical specificity and clinical relevance of our findings. Sixth, we recognize that control of three-dimensional filling of the perineural compartment is, by design, more physiological than the low-volume, layer-selective HR evaluated here. Such three-dimensional filling can produce circumferential drug distribution potentially more representative of in vivo conditions, whereas our protocol was specifically designed to isolate and characterize fluid behavior within a single anatomical layer; direct comparative studies between these two paradigms are warranted. Finally, the absence of perfusion, tissue turgor, active muscle tone, and pathological changes (e.g., inflammation or fibrosis) in our cadaveric specimens means that the observed diffusion patterns may not fully reflect in vivo conditions; thus, the spread distances reported should be regarded as provisional upper-limit estimates rather than definitive clinical values.

Detailed donor clinical information, including BMI, cause of death, and comorbidities, was not available because of the confidentiality policy of the institutional Body Donation Program. Therefore, the potential influence of donor-specific systemic conditions on the paraneural sheath or surrounding connective tissues could not be fully assessed.

The small number of specimens and the absence of a formal a priori sample size calculation also limit the generalizability of the quantitative findings. Therefore, the spread distances and circumferential grading results should be interpreted as exploratory anatomical data.

Another limitation is that dye spread along or within vascular or lymphatic structures could not be completely excluded. Although the injectate distribution was interpreted primarily as spread within the paraneural connective tissue plane based on ultrasound findings and macroscopic dissection, post-mortem tissue changes may alter tissue pressure, fluid resistance, and potential spaces. Therefore, variable spreading phenomena other than purely intra- or extra-PNS diffusion should be considered when interpreting the results.

This study quantitatively maps injectate spread within the intra-PNS, providing baseline anatomical data for future research. While previous reports have primarily focused on fascial or intramuscular injections, this study offers novel visualization and measurement of fluid spread within the intra-PNS layer.

## 5. Conclusions

This study showed that a small injection volume of 2.5 mL achieved sufficient longitudinal spread, supporting HR as a structurally selective and minimally invasive intervention. Beyond the established concept of improving interfascial gliding observed in extra-PNS HR, other mechanisms, such as the washout of pain-related biochemical mediators or the resolution of local tissue dehydration, may contribute to the therapeutic effects of intra-PNS HR. Further research is warranted to elucidate these potential pathways, including the mechanisms that remain poorly understood.

## Figures and Tables

**Table 1:** Distribution of circumferential dye spread to the side opposite the injection point within the same nerve.

Sciatic nerve	No	0%
	<2 cm	29%
	>2 cm	71%
Tibial nerve	No	0%
	<2 cm	29%
	>2 cm	71%
Common peroneal nerve	No	0%
	<2 cm	43%
	>2 cm	57%

The proportion of cases (%) showing no spread, <2 cm spread, and ≥2 cm spread on the side opposite to the injection, assessed visually after ultrasound-guided hydrorelease (2.5 mL) into the intra-paraneural sheath of the sciatic, tibial, and common peroneal nerves. No significant differences were observed among nerves.

## Data Availability

The original contributions presented in this study are included in the article. Further inquiries can be directed to the corresponding author.

## Abbreviations

The following abbreviations are used in this manuscript:

APF	Aponeurotic fascia
EPI	Epimysium
HD	Hydrodissection
HR	Hydrorelease
PNS	Paraneural sheath
